# Association of preterm birth with ADHD-like cognitive impairments and additional subtle impairments in attention and arousal malleability

**DOI:** 10.1017/S0033291717002963

**Published:** 2017-11-02

**Authors:** S.-N. James, A.-S. Rommel, C. Cheung, G. McLoughlin, D. Brandeis, T. Banaschewski, P. Asherson, J. Kuntsi

**Affiliations:** 1 King's College London, MRC Social, Genetic and Developmental Psychiatry Centre, Institute of Psychiatry, Psychology and Neuroscience, London, UK; 2 MRC Lifelong Health and Ageing Unit at University College London, London, UK; 3 Department of Child and Adolescent Psychiatry and Psychotherapy, Central Institute of Mental Health, Medical Faculty Mannheim/Heidelberg University, Mannheim, Germany; 4 Department of Child and Adolescent Psychiatry and Psychotherapy, Psychiatric Hospital, University of Zurich, Zurich, Switzerland; 5 Center for Integrative Human Physiology, University of Zurich, Zurich, Switzerland; 6 Neuroscience Center Zurich, University of Zurich, Zurich, Switzerland

**Keywords:** ADHD, adolescents, arousal, cognitive impairments, EEG, preterm birth

## Abstract

**Background:**

Whilst preterm-born individuals have an increased risk of developing attention-deficit/hyperactivity disorder (ADHD), and are reported to have ADHD-like attention and arousal impairments, direct group comparisons are scarce.

**Methods:**

We directly compared preterm-born adolescents (*n* = 186) to term-born adolescents with ADHD (*n* = 69), and term-born controls (*n* = 135), aged 11–23, on cognitive-performance, event-related potential and skin conductance level (SCL) measures associated with attention and arousal. The measures are from baseline and fast-incentive conditions of a four-choice reaction time task, previously shown to discriminate between the individuals with ADHD and controls. We aimed to establish whether preterm-born adolescents show: (a) identical cognitive-neurophysiological impairments to term-born adolescents with ADHD (b) possible additional impairments, and whether (c) the observed impairments correlate with ADHD symptom scores.

**Results:**

The preterm group, like the term-born ADHD group, showed increased mean reaction time (MRT) and reaction time variability (RTV) in the baseline condition, and attenuated contingent negative variation (CNV) amplitude (response preparation) in the fast-incentive condition. The preterm group, only, did not show significant within-group adjustments in P3 amplitude (attention allocation) and SCL (peripheral arousal). Dimensional analyses showed that ADHD symptoms scores correlated significantly with MRT, RTV and CNV amplitude only.

**Conclusions:**

We find impairments in cognition and brain function in preterm-born adolescents that are linked to increased ADHD symptoms, as well as further impairments, in lack of malleability in neurophysiological processes. Our findings indicate that such impairments extend at least to adolescence. Future studies should extend these investigations into adulthood.

## Introduction

The incidence of preterm birth (<37 weeks’ gestation) in developed countries is 5–8% (Tucker & McGuire, 2004). Whilst survival rates are improving (Goldenberg *et al.* 2008), preterm birth places an individual at an increased risk for a range of negative long-term outcomes (Bhutta *et al.* 2002; D'Onofrio *et al.* 2013). One such outcome is attention-deficit/hyperactivity disorder (ADHD) (Bhutta *et al.* 2002; Halmøy *et al.* 2012; D'Onofrio *et al.* 2013; Sucksdorff *et al.* 2015). Yet, the underlying risk pathways from preterm birth to ADHD remain poorly understood.

Individuals born preterm are also reported to have a greater risk of cognitive-neurophysiological impairments often associated with ADHD, including attention, inhibitory control, and arousal regulation difficulties (Nosarti *et al.* 2006; Aarnoudse-Moens *et al.* 2009; de Kieviet *et al.* 2012). Whilst direct comparisons between preterm-born individuals and full-term born individuals with ADHD are sparse, they can address whether the impairments reported in preterm groups are truly identical to those observed in ADHD groups or part of more wide-ranging impairments. This could help to identify biomarkers for the underlying processes linked to the increased risk for ADHD among those born preterm, and help to plan effective, targeted interventions.

A method that enables insight into the covert processes underlying observable cognitive impairments is electroencephalography (EEG). From EEG data we can extract event-related potentials (ERPs), which are electrical potentials generated by the brain in response to events, and allow the direct measurement of covert brain processes (Luck, 2005; Banaschewski & Brandeis, 2007; McLoughlin *et al.* 2014). Another informative neurophysiological method is skin conductance (SC): a simple, robust biomarker of peripheral arousal which is innervated by the sympathetic nervous system (van Lang *et al.* 2007; Boucsein *et al.* 2012).

We recently reported findings from a comparison between preterm-born adolescents and term-born ADHD adolescents on the cued continuous performance test (CPT): while we observed response preparation [the ERP index of contingent negative variation (CNV)] and response inhibition (NoGo-P3) impairments in both groups, compared witth a term-born control group, the preterm group showed an additional impairment in executive response control (GoP3), which was not associated with ADHD symptoms, suggestive of more wide-ranging neurophysiological deficits in the preterm group (Rommel *et al.* 2017). Only one other study to date, to our knowledge, has directly compared ERPs between preterm-born and ADHD groups (Potgieter *et al.* 2003). Using a visual oddball paradigm, on a small sample (*n* = 41 total), this study reported impairments [increased inhibition NoGo-N2 and increased mean reaction time (MRT), reaction time variability (RTV) and errors] only among term and preterm-born children with ADHD, compared with term-born controls and preterm-born participants without ADHD.

In addition to insight gained from neurophysiological data, another informative method, successfully applied in ADHD research, is within-task manipulations, whereby we investigate whether a specific cognitive impairment is a stable characteristic or improves under certain conditions. While increased RTV – the fluctuating speed of responding on reaction time tasks – is phenotypically and genetically strongly associated with ADHD (Kuntsi *et al.* 2010; Kuntsi & Klein, 2012; Kofler *et al.* 2013), it can improve in individuals with ADHD under certain conditions. A meta-analysis, whilst including a range of designs, demonstrated a small, though overall significant, effect of incentives on RTV (Kofler *et al.* 2013). In a four-choice reaction time task, the Fast Task, we have previously combined the effects of rewards with a faster event rate to maximize reduction of RTV, demonstrating that RTV improves significantly more in participants with ADHD than in controls (Andreou *et al.* 2007; Kuntsi *et al.* 2013). Recently, we have further measured EEG and SC simultaneously, while participants with ADHD and control participants performed the Fast Task. We found that, in the baseline (slow, unrewarded) condition, the ADHD group had impaired attentional allocation (P3 amplitude) (Cheung *et al.* 2017) and hypo-arousal [decreased skin conductance level (SCL)] (James *et al.* 2016). In the fast-incentive condition participants with ADHD improved both their P3 amplitude and SCL, more than the controls, but they now differed from controls on response preparation (CNV amplitude) (James *et al.* 2016; Cheung *et al.* 2017). These results show that although attentional allocation and hypo-arousal improved, the individuals with ADHD were not able to adjust their response preparation adequately in a changed context.

We have previously established informative ADHD-sensitive findings that emerge across the two conditions of the Fast Task when combining cognitive performance (MRT, RTV), ERP (CNV amplitude, P3 amplitude) and skin conductance (SCL) measures, which help to identify biomarkers for the underlying processes. In order to understand more about impaired brain processes in preterm-born individuals which may relate to ADHD, we now compare the data from ADHD and control participants (now including only term-born participants) to new data on identical Fast Task measures obtained from preterm-born individuals. We aim to establish, first, whether preterm-born adolescents show identical cognitive-neurophysiological impairments to those observed in term-born adolescents with ADHD. Second, we investigate whether any additional impairments are observed in the preterm group only. Third, for any impairments observed in the preterm group, we will examine their association with ADHD symptoms and clinical impairment.

## Methods

### Sample

The sample initially consisted of preterm-born participants, participants with and without ADHD and their siblings. Exclusion criteria for all groups were IQ of <70, cerebral palsy or any other medical condition that affects motor coordination including epilepsy, as well as brain disorders and any genetic or medical disorder that might mimic ADHD. In addition, preterm birth was an exclusion criterion in the ADHD and control groups, because this study aimed to establish whether the cognitive impairments associated with preterm birth reflect identical neurophysiological impairments in term-born individuals with ADHD.

The preterm group was recruited from secondary schools in Southeast England. All preterm participants had one full sibling available for ascertainment and were born before 37 weeks’ gestation. Siblings of preterm-born individuals were included in the preterm group if they were also born preterm (before 37 weeks’ gestation), to maximize the number of participants in the preterm group. Term-born siblings of preterm-born individuals were not included in this analysis. Most preterm-born participants were of European white descent (84.6%). Since here preterm birth is investigated as a potential risk factor for ADHD, preterm-born individuals who demonstrated high levels of ADHD symptoms were not excluded from the analysis (for the analysis of the sample without preterm-born individuals who met a research diagnosis for ADHD (*n* = 8), see online Supplementary Material II).

ADHD and control sibling pairs, who had taken part in our previous research (Chen *et al.* 2008; Kuntsi *et al.* 2010), were invited to take part in a follow-up study (Cheung *et al.* 2016). While ADHD-control differences for this sample have been reported previously in a study investigating ADHD impairments (James *et al.* 2016; Cheung *et al.* 2017), here the ADHD and control groups (only those who were term-born) are compared with a group of preterm-born adolescents. All participants were of white European descent and had one full sibling available for ascertainment. Participants with ADHD and their siblings were included in the ADHD group if they had a clinical diagnosis of DSM-IV combined-type ADHD during childhood and met DSM-IV criteria for any ADHD subtype at follow-up. Siblings of individuals with ADHD who did not meet DSM-IV criteria for any ADHD subtype at follow-up were not included in this analysis. The control group was initially recruited from the primary (aged 6–11 years) and secondary (aged 12–18 years) schools in the UK, aiming for an age and sex match with the ADHD sample. Control individuals and their siblings were included in the control group if they did not meet DSM-IV criteria for any ADHD subtype either in childhood or at follow-up.

The final sample consisted of 186 preterm-born participants (41 sibling pairs, 104 singletons), 69 participants with ADHD (four sibling pairs, 61 singletons) and 135 controls (61 sibling pairs, 13 singletons). The groups differed significantly in terms of age, IQ, sex distribution, GA (gestational age) and ADHD symptom scores (replicated from Rommel *et al.* 2017 in online Supplementary Material I). A 48-h ADHD medication-free period was required before assessments. Written informed consent was obtained following procedures approved by the London-Surrey Borders Research Ethics Committee (09/H0806/58) and the National Research Ethics Service Committee London—Bromley (13/LO/0068).

### Procedure

The Fast Task was administered as part of a longer assessment session at the research centre. Participants abstained from caffeine, smoking and alcohol on the day of testing. Face-to-face or telephone clinical interviews were administered to the parent of each ADHD proband shortly before or after the participant's assessment.

### Measures

#### ADHD diagnosis

The Diagnostic Interview for ADHD in Adults (DIVA) (Kooij & Francken, 2007) is a semi-structured interview designed to evaluate the DSM-IV criteria for both adult and childhood ADHD symptoms and impairment. It consists of 18 items used to define the DSM-IV symptom criteria for ADHD. The Barkley's functional impairment scale (BFIS) (Barkley & Murphy, 2006) is a 10-item scale used to assess the levels of functional impairments commonly associated with ADHD symptoms.

In the preterm and ADHD groups, ADHD was assessed using parental ADHD symptom ratings on the DIVA and the BFIS. A research diagnosis of ADHD was made if participants scored six or more on either the inattention or hyperactivity-impulsivity subscales of the DIVA and if they received two or more positive scores on two or more areas of impairment on the BFIS.

#### ADHD symptoms

For all groups, parents were asked to rate the behaviour of each sibling using the Revised Conners’ Parent Rating Scale (CPRS-R) (Conners *et al.* 1998).

#### IQ

The vocabulary and block design subtests of the Wechsler Abbreviated Scale of Intelligence Fourth Edition (WASI-IV) (Wechsler, 1999) were administered to all participants to derive estimates of IQ.

#### The Fast Task

The slow-unrewarded (baseline) condition consists of 72 trials, which followed a standard warned four-choice RT task. Four empty circles (warning signals, arranged horizontally) first appeared for 8 s, after which one of them (the target) was coloured in. Participants were asked to press the response key that directly corresponded to the position of the target stimulus. Following a response, the stimuli disappeared from the screen and a fixed inter-trial interval of 2.5 s followed. Speed and accuracy were emphasized equally in the task instructions. A comparison condition of 80 trials with a fast event rate (fore-period of 1 s) and incentives followed the baseline condition (Andreou *et al.* 2007). The fast-incentive condition is always administered after the baseline condition. Cognitive-performance measures obtained from the Fast Task include MRT (mean latency of response after target onset in milliseconds), RTV (standard deviation of target reaction time) from correct trials. Due to the longer fore-period in the slow condition, the two conditions were not matched on task length, but were matched on the number of trials. We analysed cognitive-neurophysiological performance on both the full slow condition and between three 4-min length-matched segments (results are available upon request) (Andreou *et al.* 2007).

#### EEG recording and preprocessing

The EEG was recorded from 62 channels DC-coupled recording system (extended 10–20 montage), with a 500 Hz sampling rate, impedances kept under 10 kΩ, and FCz as the recording reference electrode. The electro-oculograms (EOGs) were recorded from electrodes above and below the left eye and at the outer canthi. The EEG data were analysed using Brain Vision Analyzer (2.0) (Brain Products, Germany). After down-sampling the data to 256 Hz, the EEG data were re-referenced to the average and filtered offline with digital band-pass (0.1–30 Hz, 24 dB/oct) Butterworth filters. Ocular artefacts were identified from the data using Independent Component Analysis (ICA (Jung *et al.* 2000)). The extracted independent components were manually inspected and ocular artefacts were removed by back-projection of all but those components. All ERP averages contained at least 20 artefact-free segments. P3 amplitude was analysed as the area amplitude measure (μV × ms) at Pz between 250 and 450 ms, to reduce bias due to the varying noise levels induced by the different task conditions (Luck, 2005). For the P3 analyses, all the accepted trials were baseline-corrected using a pre-stimulus baseline of 200 ms. The mean amplitudes of this pre-target period (−200–0 ms), using a technical zero baseline as in previous CNV work (Banaschewski *et al.* 2003; Albrecht *et al.* 2013) at Cz were also analysed separately as a CNV measure (Cheung *et al.* 2017).

#### Skin conductance

SC data were measured by attaching a pair of silver–silver chloride electrodes on the thenar eminence and hypothenar eminence of participants’ non-dominant hand. SC was recorded using PSYCHLAB SC5 24 bit system (PSYCHLAB, London, UK). Stimulus onset and participant response events were recorded on a common timeline, which enabled SC activity to be stimulus-locked. SC data values were calculated using a SC system, which is based on an SC sigmoid-exponential model that allows the tonic measure of SCL to be disentangled from phasic, stimulus-associated, SC responses (SCR), and further allows the decomposition of overlapping SCRs (Lim *et al.* 1997; Williams *et al.* 2001; Figner & Murphy, 2011; Boucsein *et al.* 2012). This system, therefore, is appropriate to use in conditions with long and short inter-stimulus intervals (Williams *et al.* 2000; James *et al.* 2016, 2017). The mean of SCL was calculated per participant, across each condition.

### Statistical analyses

Regression-based corrections for age were applied to raw scores and residual scores were analysed. MRT, RTV and SCL data were skewed and transformed using the optimized minimal skew (lnskew0) in STATA version 11.1 (Stata Corporation, College Station, TX). All analyses controlled for gender, but we additionally reran analyses on a male-only subsample (online Supplementary Material II). In addition, we reran all analyses on an age-matched subsample (aged between 14 and 19 years) due to the significant group mean differences in age and the possibility of age effects on ERP measures (online Supplementary Material II). All analyses were also re-run with IQ as an additional covariate (online Supplementary Material II). Data were analysed using random intercept models in STATA, to control for non-independence of the data (i.e. data coming from siblings of one family), using a ‘robust cluster’ to estimate standard errors (Wood *et al.* 2009; Tye *et al.* 2012). *Post-hoc* analyses were reported for variables which showed a trend-like group-by-condition interaction (*p* < 0.1) . We investigated if groups differed in the slope from the baseline to fast-incentive condition, by controlling for differences in the baseline condition, indexing the degree of change. To investigate if the impairments observed in the preterm group are related to ADHD symptoms and clinical impairment, Pearson correlations were calculated between the cognitive-neurophysiological measures showing impairment in the preterm group and ADHD symptom scores and ADHD-related impairment. Correlations were run for impairments observed in the baseline condition, fast-incentive condition, and the slope from the baseline to the fast-incentive condition. If impairments were observed in both the baseline and fast-incentive condition for the same variable, correlations were run using the baseline condition only – which is more sensitive to ADHD (Kuntsi *et al.* 2013), in order to reduce the number of statistical comparisons.

## Results

The results for comparisons involving the preterm group are new and the focus here, but, for ease of comparison and completeness, we also report the statistics from the ADHD-control comparisons (previously reported for the full sample in (James *et al.* 2016; Cheung *et al.* 2017) for RTV, P3, CNV and SCL).

### Cognitive performance measures

**Fig. 1.**
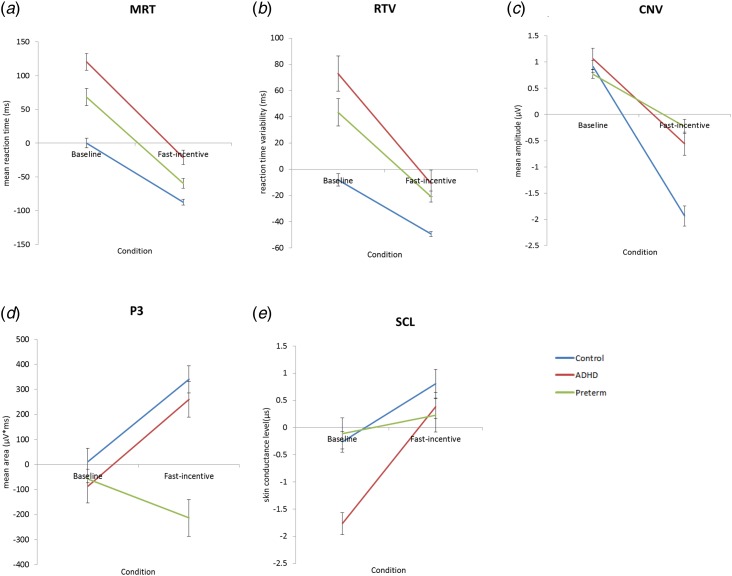
Average age regressed scores across the baseline and fast-incentive conditions of the Fast Task in the following measures: (*a*) mean reaction time = MRT (*b*) reaction time variability = RTV (*c*) contingent negative variation amplitude = CNV (*d*) P3 amplitudes and (*e*) skin conductance level = SCL. The preterm group is shown in green, attention-deficit/hyperactivity disorder (ADHD) group shown in red and the control group shown in blue. Data from ADHD and control participants in the full sample have already been presented for RTV, CNV, P3 and SCL (James *et al.* 2016; Cheung *et al.* 2017), but for ease of comparison, results specific to this analysis (ADHD and control term-born subsample) have been replicated here with the additional preterm group.

For MRT data in all groups (Fig. 1*a*), a random intercept model indicated a significant main effect of condition (*z* = −31.04, *p* < 0.01) and a main effect of group (*z* = 1.98, *p* < 0.05), but no significant group-by-condition interaction (*z* = −1.06, *p* = 0.29). The within-group difference in MRT from the baseline to the fast-incentive condition was significant in the term-born ADHD (*t* = −11.75, *p* < 0.01)and control group (*t* = −16.18, *p* < 0.01). Within-group difference in MRT was also significant in the preterm group (*t* = −13.53, *p* < 0.01). Compared with the term-born control group, the slope in MRT, indexing the extent of change from the baseline to fast-incentive condition, was significantly greater in the term-born ADHD group (*t* = 2.90, *p* < 0.01). For the preterm group, the slope in MRT was not significantly different compared with the term-born ADHD group (*p* = −1.37, *p* = 0.17), but was significantly greater compared with the term-born control group (*t* = 1.78, *p* < 0.05) (Table 2).

For RTV data for all groups (Fig. 1*b*), a random intercept model indicated a significant main effect of condition (*z* = −13.40, *p* < 0.01), a main effect of group (*z* = 3.40, *p* < 0.01) and a significant group-by-condition interaction (*z* = −2.05, *p* < 0.05). Similar to previous analyses (Cheung *et al.* 2017), compared with the term-born control group, the term-born ADHD group showed significantly greater RTV in the baseline (*t* = 3.42, *p* < 0.01) and fast-incentive (*t* = 2.58, *p* < 0.01) conditions. The within-group difference in RTV from the baseline to fast-incentive condition was significant in the term-born ADHD (*t* = −6.23, *p* < 0.01) and term-born control (*t* = −11.06, *p* < 0.01) groups, and the slope in RTV was significantly greater in the term-born ADHD group (*t* = 2.89, *p* < 0.01) compared with the term-born control group.

The preterm group, in the baseline condition, showed significantly decreased RTV compared with the term-born ADHD group (*t* = −2.05, *p* < 0.05), but significantly increased RTV compared with the term-born control group (*t* = 3.68, *p* < 0.01) (Table 1). In the fast-incentive condition, the preterm group did not differ in RTV compared with the term-born ADHD group (*t* = −1.36, *p* = 0.18), but showed significantly increased RTV compared with the term-born control group (*t* = 5.38, *p* < 0.01). The within-group difference in RTV was significant in the preterm group (*t* = −6.01, *p* < 0.01). The slope in RTV in the preterm group was, at a trend level of significance, less steep compared with the term-born ADHD group (*t* = −1.82, *p* = 0.07), but was significantly greater than in the term-born control group (*t* = 2.52, *p* < 0.05) (Table 2).

**Table 1.** Cognitive and neurophysiological measures from the baseline and fast-incentive conditions of the Fast Task: means, standard deviation (s.d.) and effect sizes (Cohen's d) for the preterm, ADHD and control groups

Variables	Condition	Preterm (*n* = 186)		ADHD (*n* = 69)		Control (*n* = 135)		Cohen's *d* effect size		
		Mean	s.d.	Mean	s.d.	Mean	s.d.	a	b	c
MRT	Baseline	594.5 (68.3)	166.3 (163.5)	616.8 (120.3)	119.1 (116.2)	530.1 (27.1)	94.0 (91.1)	0.34**	0.94**	0.30*
	Fast-incentive	466.8 (−59.4)	95.7 (93.1)	475.2 (−21.2)	95.3 (100.3)	415.7 (−87.3)	55.5 (56.8)	0.46*	0.89**	0.35**
RTV	Baseline	161.7 (43.3)	143.2 (142.3)	175.9 (72.9)	110.4 (111.0)	98.3 (−8.0)	55.9 (55.0)	0.22*	1.03**	0.46**
	Fast-incentive	97.6 (−20.7)	57.7 (57.3)	92.2 (−10.8)	80.4 (84.2)	57.1 (−49.3)	22.4 (22.9)	0.14	0.74**	0.64**
CNV (Cz)	Baseline	0.0 (0.7)	1.1 (1.16)	0.0 (1.0)	1.6 (1.6)	−0.1 (0.9)	1.3 (1.3)	0.02	0.10	0.11
	Fast-incentive	−1.0 (−0.2)	1.8 (1.8)	−1.6 (−0.5)	1.9 (1.8)	−2.9 (−1.9)	2.2 (2.2)	0.16	0.67*	0.85*
P3 (Pz)	Baseline	1038.9 (−86.8)	954.1 (105.1)	1017.5 (−68.0)	567.3 (67.0)	1190.1 (63.6)	627.8 (53.2)	0.02	0.64*	0.14
	Fast-incentive	912.5 (−213.3)	1001.2 (73.4)	1379.8 (242.0)	601.7 (71.4)	1455.4 (359.7)	630.0 (53.7)	0.44*	0.17	0.69*
SCL	Baseline	4.9 (−0.1)	3.9 (3.8)	2.8 (−1.7)	1.6 (1.6)	4.4 (−0.2)	2.2 (2.2)	0.49*	0.73*	0.04
	Fast-incentive	5.3 (0.2)	4.2 (4.2)	4.9 (0.3)	2.1 (2.1)	5.5 (0.8)	3.1 (3.0)	0.04	0.15	0.15

*p < 0.05, **p < 0.01; a=ADHD *v.* Preterm: b=ADHD *v.* Control: c=Preterm *v.* Control; ERP, event related potential; ADHD, attention-deficit/ hyperactivity disorder; MRT, mean reaction time in milliseconds; RTV, reaction time variability in milliseconds; CNV, contingent negative variation; SCL, skin conductance level.

*Note:* Values represent raw scores. Regression-based corrections in parentheses. Whilst comparisons between ADHD and control participants in the full sample have already been presented for RTV, CNV, P3 and SCL (James *et al.* 2016; Cheung *et al.* 2017) for ease of comparison, results specific to this analysis (ADHD and control term-born subsample) have been replicated here with the additional preterm group.

**Table 2.** Means and post-hoc group tests in the slope generated from plotting the baseline and fast-incentive condition of cognitive performance, ERP and skin conductance measures

	Means and 95% confidence intervals (CI)			*Post-hoc* group comparisons					
	Preterm (*n* = 186)	ADHD (*n* = 69)	Controls (*n* = 135)	a		b		c	
	Mean (95% CI)	Mean (95% CI)	Mean (95% CI)	*t*	*p*	*t*	*p*	*t*	*p*
MRT slope	−125.95	−143.24	−114.21	−1.37	0.17	2.90	<0.01	1.78	0.04
	(−137.75 to −114.14)	(−161.49 to −124.99)	(−122.62 to −105.78)						
RTV slope	−62.47	−85.54	−41.15	−1.82	0.07	2.89	<0.01	2.52	<0.01
	(−75.89 to −49.05)	(−104.67 to −66.41)	(−46.26 to −36.05)						
CNV slope (Cz)	−0.95	−1.58	−2.92	2.54	<0.01	3.12	<0.01	−7.52	<0.01
	(−1.19 to −0.70)	(−1.96 to −1.21)	(−3.25 to −2.59)						
P3 slope (Pz)	−135.34	253.58	327.77	−2.72	<0.01	−0.41	0.68	−4.05	<0.01
	(−266.17 to −4.52)	(150.97 to 356.20)	(249.76 to 405.78)						
SCL slope	0.41	2.18	1.07	−2.62	<0.01	2.60	<0.01	−1.89	0.04
	(−0.23 to 1.05)	(1.87 to 2.48)	(0.76 to 1.38)						

a=ADHD *v.* Preterm: b=ADHD *v.* Control: c=Preterm *v.* Control; ERP=event related potential; ADHD, attention-deficit/hyperactivity disorder; MRT, mean reaction time in milliseconds; RTV, reaction time variability in milliseconds; CNV, contingent negative variation; SCL, skin conductance level.

95% confidence intervals are indicated in brackets.

*Note:* Mean values represent slope values from regression-based corrections. Whilst comparisons between ADHD and control participants in the full sample have already been presented for RTV, CNV, P3 and SCL (James *et al.* 2016; Cheung *et al.* 2017), for ease of comparison, results specific to this analysis (ADHD and control term-born subsample) have been replicated here with the additional preterm group.

### ERP measures

For CNV amplitude for all groups (Fig. 1*c*), a random intercept model indicated a significant main effect of condition (*z* = −16.61, *p* < 0.01), a significant main effect of group (*z* = 3.47, *p* < 0.01) and a significant group-by-condition interaction (*z* = 9.19, *p* < 0.01). Similar to previous analyses (Cheung *et al.* 2017), compared with the term-born control group, the term-born ADHD group showed no group difference in CNV amplitude in the baseline condition (*t* = 1.24, *p* = 0.22), but showed significantly reduced CNV amplitude in the fast-incentive condition (*t* = 4.10, *p* < 0.01). The within-group difference in CNV amplitude from the baseline to fast-incentive condition was significant in both the term-born ADHD (*t* = −6.98, *p* < 0.01) and term-born control (*t* = −10.55, *p* < 0.01) group, with a significantly less steep CNV slope in the term-born ADHD group (*t* = −3.12, *p* < 0.01) compared with the term-born control group.

**Fig. 2.**
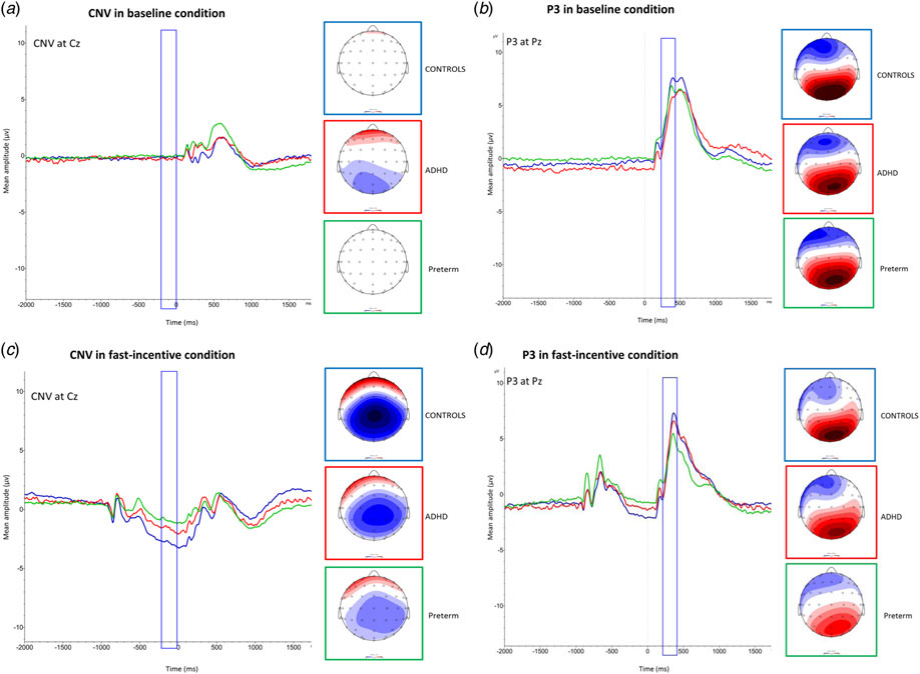
Group grand averages and topographic maps of the contingent negative variation (CNV) amplitude at the Cz electrode (shown on the left), and of P3 amplitudes at Pz electrode (shown on the right), in both the baseline (*a* and *b*) and fast-incentive conditions (*c* and *d*) of the Fast Task. The preterm group is shown in green, attention-deficit/hyperactivity disorder (ADHD) group shown in red and the control group shown in blue. Data from ADHD and control participants in the full sample have already been presented for RTV, CNV, P3 and SCL (James *et al.* 2016; Cheung *et al.* 2017), but for ease of comparison, results specific to this analysis (ADHD and control term-born subsample) have been replicated here with the additional preterm group.

The preterm group, in the baseline condition, showed no group difference in CNV amplitude compared with the term-born ADHD group (*t* = −1.48, *p* = 0.14) or the term-born control group (*t* = −0.83, *p* = 0.41) (Table 1, Fig. 2*a*). In the fast-incentive condition, the preterm group was not significantly different compared with the term-born ADHD group (*t* = 0.98, *p* = 0.33), but had a significantly reduced CNV amplitude compared with the term-born control group (*t* = 5.89, *p* < 0.01) (Table 1, Fig. 2*c*). The within-group difference in CNV amplitude from the baseline to fast-incentive condition was significant in the preterm (*t* = −5.59, *p* < 0.01) group. The slope in CNV amplitude in the preterm group was significantly less steep compared with both the term-born ADHD (*t* = −2.54, *p* < 0.01) and control (*t* = −7.52, *p* < 0.01) groups (Table 2).

For P3 amplitude for all groups (Fig. 1*d*), a random intercept model indicated a significant main effect of condition (*z* = 2.01, *p* < 0.05), a main effect of group (*z* = −3.43, *p* < 0.01) and a significant group-by-condition interaction emerged (*z* = −5.46, *p* < 0.01). Similar to previous analyses (Cheung *et al.* 2017), compared with the term-born control group, the term-born ADHD group showed significantly decreased P3 amplitude in the baseline condition (*t* = 2.62, *p* < 0.01), but they did not differ in the fast-incentive condition (*t* = 1.61, *p* = 0.14) (Table 1, Fig. 2*b*). The within-group difference in P3 amplitude from the baseline to fast-incentive condition was significant in the term-born ADHD (*t* = −3.96, *p* < 0.01) and term-born control (*t* = −6.44, *p* < 0.01) group. The slope in P3 amplitude did not differ between the term-born ADHD and control group (*t* = −0.41, *p* = 0.68).

The preterm group in the baseline condition was not significantly different in P3 amplitude compared with either the term-born ADHD (*t* = −0.34, *p* = 0.73) or control (*t* = −0.74, *p* = 0.46) group. In the fast-incentive condition, the preterm group showed significantly decreased P3 amplitude compared both to the term-born ADHD (*t* = −3.04, *p* < 0.01) and term-born control (*t* = −5.26, *p* < 0.01) groups (Table 1, Fig. 2*d*). The within-group difference in P3 amplitude from the baseline to fast-incentive condition was not significant in the preterm group (*t* = −1.57, *p* = 0.16). The slope in P3 amplitude in the preterm group was less steep compared with both the term-born ADHD (*p* = −2.72, *p* < 0.01) and term-born control (*t* = −4.05, *p* < 0.01) groups (Table 2).

### SC measures

For SCL for all groups (Fig. 1*e*), a random intercept model indicated a significant main effect of condition (*z* = −5.74, *p* < 0.01), but no main effect of group (*z* = 0.02, *p* = 0.99), and a trend towards a group-by-condition interaction (*z* = −1.68, *p* = 0.09). Similar to previous analyses (James *et al.* 2016), the term-born ADHD group showed significantly decreased SCL compared with the term-born control group in the baseline condition (*t* = −4.55, *p* < 0.01), but not in the fast-incentive condition (*t* = 0.91, *p* = 0.36). The within-group difference in SCL from the baseline to fast-incentive condition was significant in the term-born ADHD (*t* = 9.29, *p* < 0.01) and term-born control (*t* = 4.85, *p* < 0.01) groups. The slope in SCL was significantly steeper in the term-born ADHD group compared with the term-born control group (*t* = 2.60, *p* < 0.05).

The preterm group, in the baseline condition, showed significantly increased SCL compared with the term-born ADHD group (*t* = 4.01, *p* < 0.01), but did not differ from the term-born control group (*t* = 0.30, *p* = 0.76). In the fast-incentive condition, the preterm group was not significantly different compared with the term-born ADHD group (*t* = −0.10, *p* = 0.91) or compared with the term-born control group (*t* = −1.02, *p* = 0.31). The within-group difference in SCL from the baseline to fast-incentive condition was not significant in the preterm group (*t* = 0.83, *p* = 0.41). The slope in SCL in the preterm group was less steep compared with both the term-born ADHD (*p* = −2.62, *p* < 0.01) and term-born control (*t* = −1.89, *p* < 0.05) groups (Table 2).

Excluding the eight preterm-born individuals meeting diagnostic criteria for a research diagnosis of ADHD, using a male-only sample, using an age-match subsample or re-running the analysis with IQ as a covariate (online Supplementary Material II), did not change the significance of the results.

### Associations with ADHD symptoms and impairment

Correlations were run in the preterm group (*n* = 186) to investigate if the cognitive-neurophysiological differences observed in the preterm group, compared with term-born controls, are related to ADHD symptoms and ADHD-related clinical impairments. In order to reduce the number of statistical comparisons, correlations were run using the baseline condition only - which is more sensitive to ADHD (Kuntsi *et al.* 2013) – if impairments were observed in both the baseline and fast-incentive condition for the same variable. In the preterm group, baseline performance of MRT and RTV, and the slope of MRT and RTV, were significantly correlated with ADHD symptoms and ADHD impairment (Table 3). CNV amplitude in the fast-incentive condition was correlated with ADHD symptoms and ADHD impairment, but the correlation with the slope in CNV amplitude did not reach significance (Table 3). P3 amplitude in the fast-incentive condition, the slope in P3 amplitude, and the slope in SCL, were not significantly correlated with ADHD symptoms or ADHD impairment (Table 3).

**Table 3.** Pearson correlations (two-tailed) between cognitive-neurophysiological impairments observed in the preterm group with interview-based ADHD symptoms and clinical impairment within the preterm group only (n=186)

	ADHD symptoms		Impairment	
	*r*	*p*	*r*	*p*
MRT Baseline	0.23	<0.01	0.19	<0.01
RTV Baseline	0.24	<0.01	0.20	<0.01
CNV Fast-incentive	0.15	0.04	0.13	0.05
CNV slope	0.08	0.28	0.06	0.41
P3 Fast-incentive	−0.10	0.17	−0.09	0.17
P3 slope	−0.06	0.41	−0.12	0.10
SCL slope	−0.08	0.22	−0.11	0.14

Baseline, Baseline condition; Fast-incentive, Fast incentive condition; slope, the slope generated from plotting performance from the baseline to fast-incentive condition. ADHD, attention-deficit/ hyperactivity disorder; DIVA, Diagnostic Interview for ADHD in Adults; MRT, mean reaction time in milliseconds; RTV, reaction time variability in milliseconds; CNV, contingent negative variation amplitude at Cz; P3, P3 amplitude at Pz; SCL, skin conductance level.

*Note:* In order to reduce the number of statistical comparisons, correlations were run using the baseline condition only – which is more sensitive to ADHD – if impairments were observed in both the baseline and fast-incentive condition for the same variable.

## Discussion

In a detailed analysis of cognitive-neurophysiological processes during RT performance under baseline and fast-incentive conditions, we provide evidence, first, for ADHD-like impairments in adolescents born preterm in speed and variability of reaction times (MRT and RTV in baseline condition) and in response preparation (CNV in fast-incentive condition). These findings from group comparisons were further confirmed by within-group analyses that showed how each of these impairments correlated with the continuum of ADHD symptoms (and impairments) in individuals born preterm. Second, the adolescents born preterm did not show ADHD-like impairments in the ERP index of attention allocation (P3) or skin conductance-measured arousal (SCL) in the baseline condition, but were unlike either the ADHD or control group in showing an unusual lack of malleability in P3 amplitude and SCL from baseline to fast-incentive condition, indicating a lack of malleability in attention allocation and arousal from the baseline to fast-incentive condition in the individuals born preterm. Overall, we show how specific impairments in cognitive and brain function observed among preterm-born individuals relate to their increased ADHD symptoms, whereas their additional impairments were not significantly associated with ADHD symptoms. Our findings provide further evidence, in line with other studies, that preterm birth is a risk factor for developing some ADHD-related cognitive-neurophysiological impairments (Aarnoudse-Moens *et al.* 2009). However, our findings also indicate further, non-ADHD related, impairments, indicating there are differentiating neurophysiological processes in preterm individuals. Given that the last trimester is crucial for growth and development of brain networks (Johnson, 2003; Ball *et al.* 2014), it is feasible that giving birth prematurely could disrupt this process and result in aberrant networks associated with ADHD, and with further impairments.

Our finding that the ERP-index of response preparation (CNV) shows an ADHD-like impairment in adolescents born preterm replicates our previous CNV finding on the CPT in the same sample (Rommel *et al.* 2017). These observations are in line with previous evidence of abnormalities in response preparation in children born preterm (Mikkola *et al.* 2007, 2010; Hövel *et al.* 2014), and we now show how these impairments are linked to the increased ADHD symptoms in individuals born preterm. The further ADHD-like impairments we observed in the preterm-born group in the speed and variability of reaction times (MRT and RTV) were significantly milder among the preterm-born than in individuals with ADHD, although both groups significantly differed from controls. In our previous analysis on CPT data on the same sample, we did not observe differences in MRT and RTV between the preterm and controls groups (Rommel *et al.* 2017), suggesting that the milder MRT and RTV impairments in individuals born preterm may only be observed in tasks that show particularly strong impairments in individuals with ADHD. Increased MRT and RTV in preterm-born children have also been reported for a visual oddball task (Potgieter *et al.* 2003), and an attention network test study reported increased lapses of attention in preterm-born individuals (de Kieviet *et al.* 2012). We now show how the increased MRT and RTV in individuals born preterm, similar to attenuated CNV, are related to their increased ADHD symptoms.

While the above findings point to specific ADHD-like impairments in cognition and brain function, our further findings on attention allocation (P3) and peripheral arousal (SCL) indicate that preterm birth is associated with only some, and not all, impairments seen in ADHD, as well as with further unique impairments not associated with ADHD. The adolescents born preterm did not show the ADHD-like impairment in attention allocation (P3) and peripheral hypo-arousal (SCL) in the baseline condition. Yet subtle impairments in P3 amplitude and SCL were observed in the preterm group in the lack of adjustment and malleability from the baseline to fast-incentive condition that are seen in the other groups. For response preparation (CNV), both preterm and ADHD groups showed the reduced change between task conditions, compared with controls, but the lack of adjustment was significantly stronger for the preterm than term-born ADHD group. Overall, the reduced neurophysiological sensitivity to the effects of incentives and a faster event rate in the individuals born preterm is intriguing, calling for a further investigation in future research.

A limitation of our study is the small sample of females in the ADHD group (*n* = 8): whilst we controlled for gender, we could not directly examine sex differences between the groups. We were also unable to investigate whether risk factors for being born preterm (e.g. poverty, malnutrition) might account for the findings in our sample. We show, however, that the impairments are not due to IQ, as controlling for IQ did not change the results.

In conclusion, our investigation of preterm-born adolescents indicates both impairments in cognition and brain function that are linked to increased ADHD symptoms as well as further, subtle impairments in lack of malleability in specific neurophysiological processes. We show how such impairments in individuals born preterm extend to at least adolescence, even in a well-functioning sample recruited from mainstream schools. Greater awareness of the risk of developing ADHD-like and wider-ranging impairments in preterm-born individuals could lead to earlier identification and intervention strategies. Future studies should extend these investigations into adulthood.

## Supplementary material

For supplementary material accompanying this paper visit https://doi.org/10.1017/S0033291717002963. click here to view supplementary material
