# Supplementary material for: Association of preterm birth with ADHD-like cognitive impairments and additional subtle impairments in attention and arousal malleability
Source: Psychol Med. 2017 Nov 2;48(9):1484–93. doi: 10.1017/S0033291717002963 (PMC6088527; doi:10.1017/S0033291717002963)
Supplement: Supplementary file 1 [file S0033291717002963sup001.docx]

# SUPPLEMENTARY MATERIALS

**Table 1: Descriptive statistics.** This information has already been reported (Rommel et al. under review), but for ease of comparison, results have been replicated here.

**Supplementary Material I: Descriptive statistics. This information has already been reported (Rommel et al. under review), but for ease of comparison, results have been replicated here.**

*Note:* ADHD=attention-deficit/ hyperactivity disorder; GA=gestational age; SD=standard deviation, BFIS= Barkley Functional Impairment Scale.

|  | **Preterm** | **ADHD** | **Control** | **Statistic** | **df** | **p-value** |
| --- | --- | --- | --- | --- | --- | --- |
|  | n=186 | n=69 | n=135 | - | - | - |
| **GA in weeks (SD)** | 33.0 (3.0) | 39.9 (1.4) | 39.9 (1.3) | t=-23.0 | 253 | <0.01 |
| **IQ (SD)** | 104.7 (12.3) | 97.7 (13.8) | 110.4 (12.2) | t=-3.2 | 253 | 0.02 |
| **Age (SD)** | 14.9 (1.9) | 18.5 (3.0) | 17.8 (2.1) | t=-12.0 | 253 | <0.01 |
| **Age range** | 11.0-20.0 | 12.7-25.9 | 11.9-21.6 | - | - | - |
| **Males %** | 54.3 | 88.4 | 75.6 | t=4.61 | 253 | <0.01 |
| **Conners parent-rated ADHD symptom score (SD)** | 11.2 (9.4) | 35.8 (10.6) | 7.0 (5.6) | t=1.97 | 253 | 0.05 |
| **BFIS score (SD)** | 3.7 (4.1) | 16.4 (5.4) | 2.1 (2.5) | t=-2.23 | 253 | 0.03 |

**Supplementary Material II: Effect sizes for group comparisons in the baseline and fast-incentive condition: a) ADHD vs control b) Preterm vs ADHD c) Preterm vs control differences.**

|  | **Condition** | **Model 1 (shown in paper):**  covariate – sex;  Full sample | | | **Model 2:**  Covariate – sex; Exclude preterm with ADHD | | | **Model 3:**  covariate – sex;  Male-only sample | | | | **Model 4:**  covariate – sex;  Age-matched sample | | | | **Model 5:**  covariate – sex and IQ;  Full sample | | |
| --- | --- | --- | --- | --- | --- | --- | --- | --- | --- | --- | --- | --- | --- | --- | --- | --- | --- | --- |
|  |  | Cohen’s d effect size | | | Cohen’s d effect size | | | Cohen’s d effect size | | | | Cohen’s d effect size | | | | Cohen’s d effect size | | |
|  |  | a | b | c | a | b | c | a | b | c | a | | b | c | a | | b | c |
| **MRT** | Baseline | 0.94** | 0.34** | 0.30* | 0.94** | 0.37** | 0.24* | 1.04** | 0.41* | 0.25 | 0.91** | | 0.40** | 0.20* | 1.04** | | 0.45** | 0.26* |
|  | Fast-incentive | 0.89** | 0.46* | 0.35** | 0.89** | 0.41* | 0.34** | 0.83** | 0.42* | 0.32** | 0.69** | | 0.29* | 0.34** | 0.89** | | 0.40* | 0.35** |
| **RTV** | Baseline | 1.03** | 0.22* | 0.46** | 1.03** | 0.29* | 0.46** | 1.05** | 0.24* | 0.43** | 1.28** | | 0.22* | 0.40** | 1.15** | | 0.31* | 0.45** |
|  | Fast-incentive | 0.74** | 0.14 | 0.64** | 0.74** | 0.13 | 0.47** | 0.68** | 0.18 | 0.57** | 0.82** | | 0.17 | 0.66** | 0.73** | | 0.15 | 0.63** |
| **CNV** | Baseline | 0.10 | 0.02 | 0.11 | 0.10 | 0.03 | 0.08 | 0.17 | 0.16 | 0.12 | 0.03 | | 0.01 | 0.01 | 0.04 | | 0.16 | 0.12 |
|  | Fast-incentive | 0.67* | 0.16 | 0.85* | 0.67* | 0.18 | 0.85* | 0.61* | 0.24 | 0.86* | 0.68* | | 0.14 | 0.87* | 0.65* | | 0.18 | 0.85* |
| **P3** | Baseline | 0.64* | 0.02 | 0.14 | 0.64* | 0.02 | 0.09 | 0.41 | 0.09 | 0.08 | 0.49 | | 0.28 | 0.13 | 0.46 | | 0.03 | 0.08 |
|  | Fast-incentive | 0.17 | 0.44* | 0.69* | 0.17 | 0.41* | 0.63* | 0.2 | 0.37* | 0.59* | 0.03 | | 0.73* | 0.72* | 0.27 | | 0.44* | 0.65* |
| **SCL** | Baseline | 0.73* | 0.49* | 0.04 | 0.73* | 0.45* | 0.01 | 0.71* | 0.56* | 0.13 | 1.03* | | -0.50* | 0.19 | 0.78* | | 0.48* | 0.01 |
|  | Fast-incentive | 0.15 | 0.04 | 0.15 | 0.15 | 0.02 | 0.14 | 0.02 | 0.06 | 0.08 | 0.03 | | 0.11 | 0.14 | 0.15 | | 0.04 | 0.15 |

*Note:* Cohen effect sizes *p<0.05, **p<0.01; a=ADHD vs Control: b=ADHD vs Preterm: c=Preterm vs Control; ERP=event related potential; ADHD=attention-deficit/ hyperactivity disorder; MRT=mean reaction time in milliseconds; RTV=reaction time variability in milliseconds; CNV=contingent negative variation; SCL=skin conductance level.
